# Isochrone-based Identification of Gaps in Neurovascular Care in Germany

**DOI:** 10.1007/s00062-025-01537-0

**Published:** 2025-06-26

**Authors:** Marius Vach, Christian Rubbert, Julian Caspers, Sven G. Meuth, Marc Pawlitzki, Lars Masanneck

**Affiliations:** 1 Institute for Diagnostic and Interventional Radiology, University Hospital and Medical Faculty University Hospital Düsseldorf, Duesseldorf, Germany; 2 Department of Neurology, University Hospital and Medical Faculty University Hospital Düsseldorf, Heinrich-Heine University Duesseldorf, Moorenstraße 5, 40225 Duesseldorf, Germany

**Keywords:** Stroke, Intracranial hemorrhage, Isochrone analysis, Neurovascular disease, Germany

## Abstract

**Purpose:**

Modern endovascular techniques enable the treatment of various neurovascular diseases. Given the complexity of these interventions, a certification system was introduced to ensure standardized care at specialized treatment centers. We used a driving-time-based isochrone approach to identify care gaps in different German Society of Interventional Radiology (DeGIR) certified neurovascular treatment centers.

**Methods:**

DeGIR-certified neurovascular centers for minimally invasive stroke care (module E), neurovascular vessel anomalies (module F), and neurovascular therapy (module EF) were geocoded and driving-time-based isochrones were calculated for 30, 60, 90, and 120 min. The resulting contours were aggregated and combined with the 2025 population estimates from the Global Human Settlement Layer to estimate residents’ access.

**Results:**

The analysis identified gaps in under-60-minute reachability, notably in northeastern Germany and parts of Rhineland-Palatinate, Saarland, and the southwest, with modules EF and F most affected, while module E fared better. Within 120 min, coverage was nearly complete across all modules. On a population level, 59.4% of residents lived within 30 min, 92.81% within 60 min, and 99.98% within 120 min of a module E center. Module F reached 45.8%, 84.26%, and 99.73%, respectively, with module EF showing intermediate accessibility.

**Discussion:**

The driving-time-based isochrone approach identifies regions where access to specialized neurovascular care is limited—a critical issue in emergencies like stroke or aneurysm hemorrhage. Although immediate stroke care is generally more accessible than care for neurovascular anomalies, thrombectomy within an acceptable timeframe is not available to the entire population. These findings can guide strategies to enhance neurovascular care across Germany.

**Supplementary Information:**

The online version of this article (10.1007/s00062-025-01537-0) contains supplementary material, which is available to authorized users.

## Introduction

Modern endovascular techniques enable the effective treatment of a wide range of neurovascular diseases. Particularly, endovascular treatment of acute stroke (“mechanical thrombectomy”) has been established as an effective therapeutic intervention and has become standard of care [1–3]. Given the complexity of these interventions, a certification system was introduced to ensure standardized care at specialized treatment centers. In Germany, these centers are certified by the German Society for Interventional Radiology (DeGIR) with three distinct certificates. The certification “Module E” is awarded to specialized center for minimally invasive stroke therapy meeting certain standards, whereas “Module F”-certification marks specialized centers treating neurovascular malformations. Additionally, centers can receive an overarching certification as a center for neurovascular therapy (“Module EF”).

Since neurovascular emergencies, such as acute ischemic or hemorrhagic stroke, demand urgent specialized care, rapid access to these centers is of utmost importance [4–6]. However, the importance of access to such centers extends beyond emergency situations, as these centers also play a crucial role in aftercare, surveillance and elective procedures. Moreover, longer travel times are expected to hinder access to optimal care and may result in worse health outcomes, as shown in other medical contexts [7].

To date, no systematic analysis of the distribution and accessibility of neuroradiological interventional centers in Germany has been conducted. Therefore, we employed a drive-time-based isochrone approach to identify gaps in the provision of care for these specialized neurovascular treatment centers and correlated the resulting isochrones with population data.

## Methods

### Overview

For the analysis, we used the open-source software OpenStreetMap to calculate isochrones, i.e., areas reachable within a specified driving time from a given start location. Additionally, we estimated the population residing in the isochrone-covered areas.

### Data

The German Society for Interventional Radiology (DeGIR) issues several different certificates for radiological centers with specific neurovascular competence. Neuroradiological centers can be certified with module E (“center for minimally invasive stroke therapy”), module F (“center for neurovascular malformations”) or both (module EF, “center for neurovascular therapy”). Lists of DeGIR-certified centers were obtained from the public website of the DeGIR (https://degir.de/degir-dgnr-zentren/). The most recent data was collected on the 7th of February 2025. To check the influence on coverage of non-certified neurovascular centers, university hospitals that are not DeGIR-certified were also merged with centers certified under module E for minimally invasive stroke therapy, as we assumed these institutions likewise offer such services. We considered all full members of the German University Hospital Association (“Vollmitglieder des Verbands der Universitätsklinika Deutschlands e. V. (VUD)”) as of May 2nd, 2025 and identified a total of 5 that were merged (Bonn, Essen, Freiburg, Halle (Saale), and Heidelberg). We calculated isochrones for this merged list as a separate condition. Additionally, the total number of beds for each hospital in the dataset was obtained from the “Bundes-Klinik-Atlas” (https://bundes-klinik-atlas.de/), an official register of all German hospitals by the German Ministry of Health (Last access 3rd of May 2025).

### Isochrones

Driving-time-based isochrones are geographic contours connecting points of equal travel time by road from a given location, considering factors such as road networks, speed limits, and typical traffic conditions. These temporal-spatial boundaries represent the areas that can be reached within specific time thresholds using a particular mode of travel, such as by foot or vehicle, providing a more realistic measure of accessibility than straight-line distance. In healthcare planning, isochrones are especially valuable for analyzing population access to medical facilities and identifying potentially underserved areas based on travel time requirements.

### Data Analysis

Addresses of centers were manually reviewed for driving restrictions, which would apply to civilian traffic, but not emergency services. In case of such restrictions, addresses were slightly altered to avoid such restrictions, but still allow for correct isochrone calculation. Analyses were conducted using Python 3.8.13 (Python Software Foundation, Delaware, USA) with pandas package version 1.3.4. Geocoding and isochrone calculation services relied on data from the OpenStreetMap (OSM) project. Geospatial analysis and geometric object aggregation were performed using Rasterio (version 1.1.120) and Shapely (version 1.7.1). Address geocoding was carried out using the geopy package (version 2.2.022) with Nominatim geocoder for OSM data. Isochrones for geocoded neurovascular centers were generated for car road travel times of 30, 60, 90, and 120 min using a locally hosted instance of openrouteservice (version 7.1.0). These time points were selected as empirical analysis showed that beyond 120 min, the increase in coverage provided diminishing returns for the study’s objectives. The isochrones for each certified center for the different certificates were aggregated through a union operation to determine the full coverage area for the respective care network and travel time. The resulting geometric shape was then applied as a mask to the publicly available Global Human Settlement Population Grid R2023A(GHS-POP) [8, 9] to estimate the population residing within the covered area in 2025. The results were visualized using matplotlib (version 3.8). For sub-analysis of German states, we applied state masks to the previously calculated geometric figures and calculated the respective analyses on the level of the 16 German federal states.

### Data Availability

This study only used publicly available data. Interactive figures of the main results are available in a GitHub repository (https://entspannter.github.io/Neuroradiology-interventional-centers/interactive_map_modules_light.html).

## Results

In total we analyzed 107 module E centers (“center for minimally invasive stroke therapy”), 53 module F centers (“center for neurovascular malformations”) and 59 module EF centers (“center for neurovascular therapy”, see Table 1).

**Table 1:** Number of hospitals and total beds per DeGIR certification grouped by German federal state

DeGIR module	Module E		Module F		Module EF	
State	# of hospitals	Total beds	# of hospitals	Total beds	# of hospitals	Total beds
Baden-Württemberg	12	10,740	7	7168	7	10,727
Bayern	15	12,109	8	7939	7	7141
Berlin	8	7189	3	4796	3	4796
Brandenburg	3	1967	0	0	0	0
Bremen	1	832	1	832	1	832
Hamburg	4	3571	2	2341	2	2341
Hessen	9	7619	6	5274	6	5274
Mecklenburg-Vorpommern	3	2662	0	0	0	0
Niedersachsen	9	6166	3	2220	3	2220
Nordrhein-Westfalen	19	12,734	8	6908	8	6908
Rheinland-Pfalz	2	2228	1	1354	1	2708
Saarland	1	1227	1	1227	1	1227
Sachsen	7	6102	3	3806	3	3806
Sachsen-Anhalt	1	754	1	754	1	754
Schleswig-Holstein	7	3985	3	2247	3	2247
Thüringen	6	4730	2	2484	2	2484

**Fig. 1:**
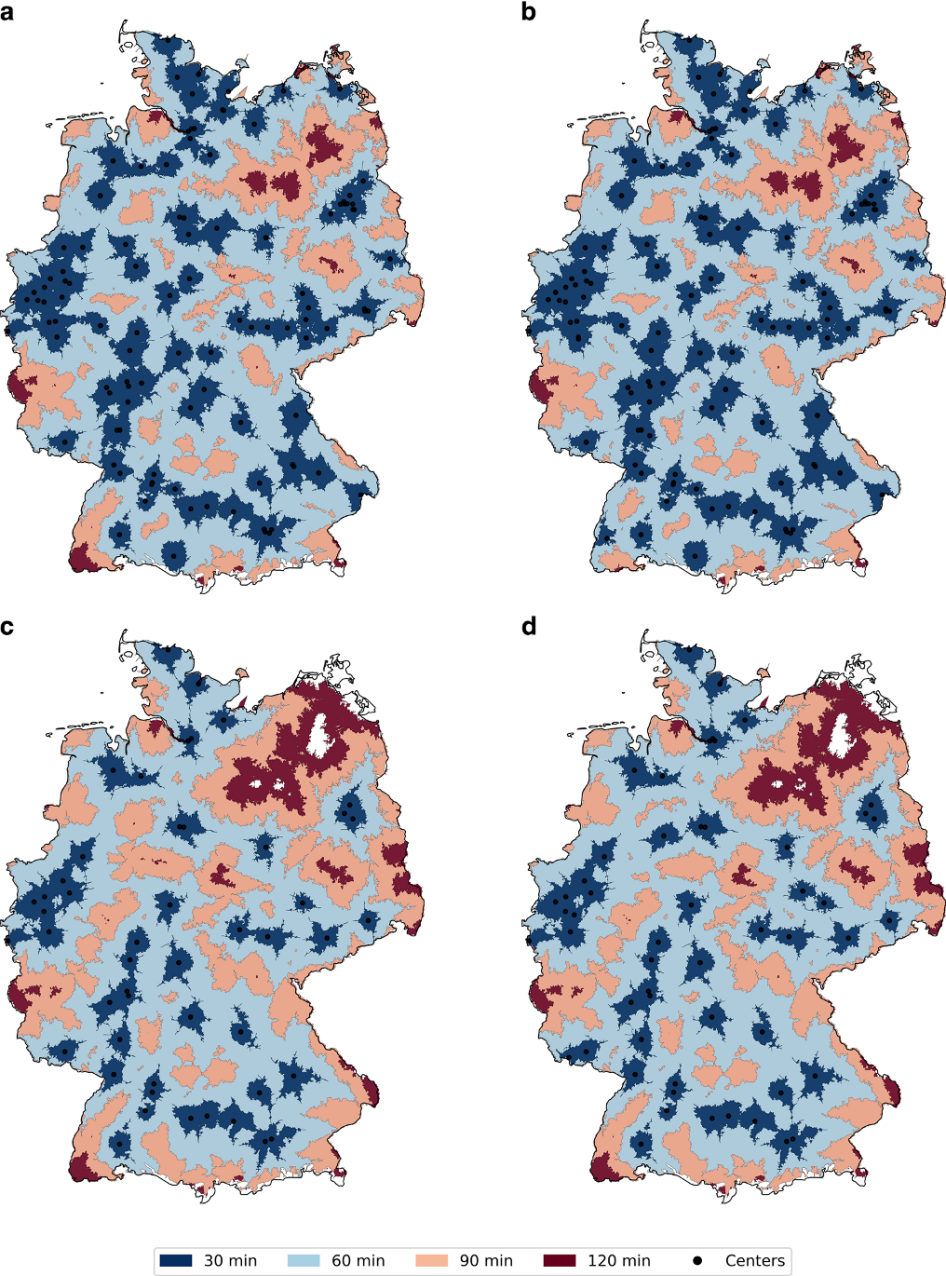
*Accessibility Drive-time Isochrones for Certified Neuroradiology Centers in Germany. *Maps depict drive-time isochrones (30, 60, 90, and 120 min) for DeGIR-certified neurovascular treatment centers in Germany. Panels show accessibility for **a** Module E (stroke therapy centers), **b** Module E and all non-certified university hospitals, which are assumed to also deliver this care (a total of 5), **c** Module F (centers for neurovascular malformations), and **d** Module E & F combined. Black dots indicate certified centers. Regions beyond 120 min highlight potential care gaps, particularly for Module F in northeastern and southwestern Germany. Corresponding coverage on a population level can be found in Table 2

The visual isochrone analysis revealed coverage gaps for neurovascular centers within a 60 min drive, particularly in the northeast of Germany as well as in parts of Rhineland-Palatinate, Saarland and the far southwest of Germany. These gaps were most pronounced for modules EF and F, whereas module E showed better coverage. Including non-certified tertiary university hospitals in the analysis slightly improved the reachability of neurovascular centers, especially in the southwest of Germany. Within a 120-minute drive, however, coverage was complete or nearly complete for all modules. Figure 1 shows the isochrone maps for each module.

A population-level analysis examining driving times to certified neurovascular therapy centers revealed high overall accessibility across Germany. For centers certified under Module E, 59.4% of the population could reach a facility within 30 min, rising to 92.8% by 60 min. Nearly complete coverage (rounded 100.0%) was achieved within 120 min. Including non-certified tertiary university hospitals in the analysis, only improved the population coverage slightly (from 59.4 to 60.7% for 30 min and from 92.8 to 94.3% for 60 min reachability), with however relevant changes in certain ares such as Baden-Württemberg (86.5 to 94.9% for 60 min). Module F‑certified centers showed slightly lower accessibility, with 45.8% of the population within 30 min and 84.3% within 60 min, reaching 99.7% coverage at 120 min. Centers with the combined Module EF certification demonstrated intermediate accessibility, with 47.8% of the population within 30 min, 86.4% within 60 min, and a rounded 100.0% within 120 min. Notably, the most substantial increases in population coverage occurred between 30- and 60-minute driving times across all certification types, with smaller incremental gains beyond 60 min. The population-level results are presented in Table 2.

**Table 2:** Isochrone-based population level accessibility of neurovascular centers in Germany

Driving Time (minutes)	Population Module E	Population Module E + University Hospitals	Population Module F	Population Module E&F
30	49,527,276 (59.4%)	50,501,791 (60.7%)	38,187,983 (45.8%)	39,764,817 (47.8%)
60	77,382,238 (92.8%)	78,469,033 (94.3%)	70,252,852 (84.3%)	71,899,960 (86.4%)
90	82,727,226 (99.2%)	83,094,383 (99.9%)	81,612,478 (97.9%)	81,657,589 (98.2%)
120	83,364,299 (100.0%)	83,375,153 (100.0%)	83,156,095 (99.7%)	83,164,485 (100.0%)

When looking at more granular population coverage by state we noticed that driving time varies dramatically between German city-states and larger territorial states. City-states such as Bremen, Hamburg and Berlin exhibit near-complete population coverage even with short driving times, whereas territorial states demonstrate pronounced heterogeneity: As an example, Hesse reaches good coverage with 60 min of driving time (99.3% (E) and 98.2% (E & F)) and even the largest state, Bavaria, achieves 93.5% coverage within 60 min for E‑certified centers and 86.2% for E & F‑certified centers. By contrast, the Western state Rhineland-Palatine attains only moderate population coverage at respective 81.5% (E) and 66.8% (E & F), while the Eastern state Mecklenburg-Western Pomerania falls further to 78.9% (E) and a mere 18.2% (E & F). Isochrone maps and more granular calculations for each German federal state can be found in the supplemental material.

## Discussion

An increasing number of neurovascular diseases is treated with modern endovascular techniques. However, many of these techniques require a high level of specialization, which led to the certification process of specialized neurovascular centers by the German Society for Interventional Radiology (DeGIR). The aim of this study was to use driving-time-based isochrone analysis to examine the distribution and accessibility of these neurovascular centers in Germany.

Our analysis identified a prominent gap in specialized neurovascular care infrastructure in the Northeastern region of Germany as well as in parts of Rhineland-Palatinate and Saarland. This gap is likely driven by sparse infrastructure, lower population density, and fewer certified centers in these rural regions. Nonetheless, such geographic inequity may delay time-sensitive interventions for stroke or other neurovascular emergencies, exacerbating health outcome disparities in regions that are already comparatively socioeconomically disadvantaged [10]. Conversely, the high population coverage within 120 min (> 99.7% for every certificate) reflects the concentration of certified centers in densely populated urban areas, aligning with Germany’s urban-centric healthcare infrastructure. However, compared to the US, where a recent study estimated that less than half of the population can reach a thrombectomy center within one hour [11, 12], neurovascular centers in Germany are more accessible to a larger proportion of the population, presumably due to the on average higher population density of Germany. Our findings in this study are consistent with similar analyses of specialized neuroinflammatory and memory centers as well as a study on the accessibility of general inpatient care in Germany [13–15]. Recent studies on healthcare facility reachability in a Chinese metropolis and in a global analysis found a trend of these facilities being located in densely populated areas, even outside the realm of specialized healthcare [16, 17]. This aligns with our finding on the distribution of stroke centers in Germany. Compared to the faster reachability of general healthcare facilities for a majority of the population reported in these studies, our analysis focused on less numerous, highly specialized endovascular stroke centers.

In stroke care, rapid access to a specialized center is crucial. A previous study estimated that saving 4 min improves the outcome for 1% of patients [6]. Our analysis indicates that in some rural areas, a neurovascular center cannot be reached within 60 min. This not only poses a significant challenge to providing state-of-the-art stroke treatment in these regions, but arguably may also result in worse patient outcomes, given the critical importance of early intervention. This issue warrants consideration in political debates on healthcare reform in Germany, especially given the context of recent legislation, which partially aims to centralize resources withing Germany’s healthcare system.

Our analysis has some limitations. For instance, acquiring or not acquiring certification may not fully reflect the actual capabilities of regional healthcare providers. For example, the extended driving times observed in southwestern Germany can be (partly) attributed to the lack of certification for institutions such as the University Hospital Freiburg or a hospital in Trier—both of which serve as major neurovascular centers involved in regional stroke care. Nevertheless, official certification is still deemed a useful surrogate marker for identifying potential gaps in care. As our analysis with all university hospitals added has shown, in most regions with limited access, however, the issue is not a lack of certification but rather the absence of any nearby neuroradiological centers.

The driving-time-based analysis has limitations in capturing the actual accessibility of neurovascular centers, particularly in remote areas and on islands, where helicopter transport is commonly used for emergency access to specialized hospitals. Additionally, the driving time calculations are based on standard driving behavior, while ambulances can sometimes drive faster, potentially causing an underestimation of some neurovascular centers’ accessibility in emergency situations. Additionally, innovative concepts like “drip and ship” or “drive the doctor” have been established in Germany to improve the acute stroke care in more rural areas. While studies have shown a comparable outcome for patients treated using “drip and ship” compared to direct transportation to a comprehensive care center [18] a big analysis of the German Stroke Register showed worse outcomes for patients treated in a “drip-and-ship” model [19]. Further studies are needed to assess in which circumstances such models are viable models for undersupplied regions.

This study provides a foundational assessment of neurovascular care accessibility in Germany. While population coverage metrics are encouraging, persistent geographic gaps call attention to ensure equitable care. Given the limited scalability of specialized neuroradiological care in peripheral regions, this study underscores the need for alternative strategies, such as telemedicine or optimized patient transfer processes, to ensure timely access, especially in critical situations. By integrating these findings into policy and infrastructure planning, Germany can ensure specialized neurovascular treatments are more widely available to a broader population, regardless of location.

## Supplementary Information

Different conditions of coverage plotted by German state

Underlying tabular representation of the data

## Data Availability

This manuscript fully relies on publicly available data. Interactive figures of the main results are available in a GitHub repository (https://entspannter.github.io/Neuroradiology-interventional-centers/interactive_map_modules.html).
