# Supplementary material for: Isochrone-based Identification of Gaps in Neurovascular Care in Germany
Source: Clin Neuroradiol. 2025 Jun 26;35(4):747–53. doi: 10.1007/s00062-025-01537-0 (PMC12552351; doi:10.1007/s00062-025-01537-0)
Supplement: Supplementary file 1 — Different conditions of coverage plotted by German state [file 62_2025_1537_MOESM1_ESM.pdf]

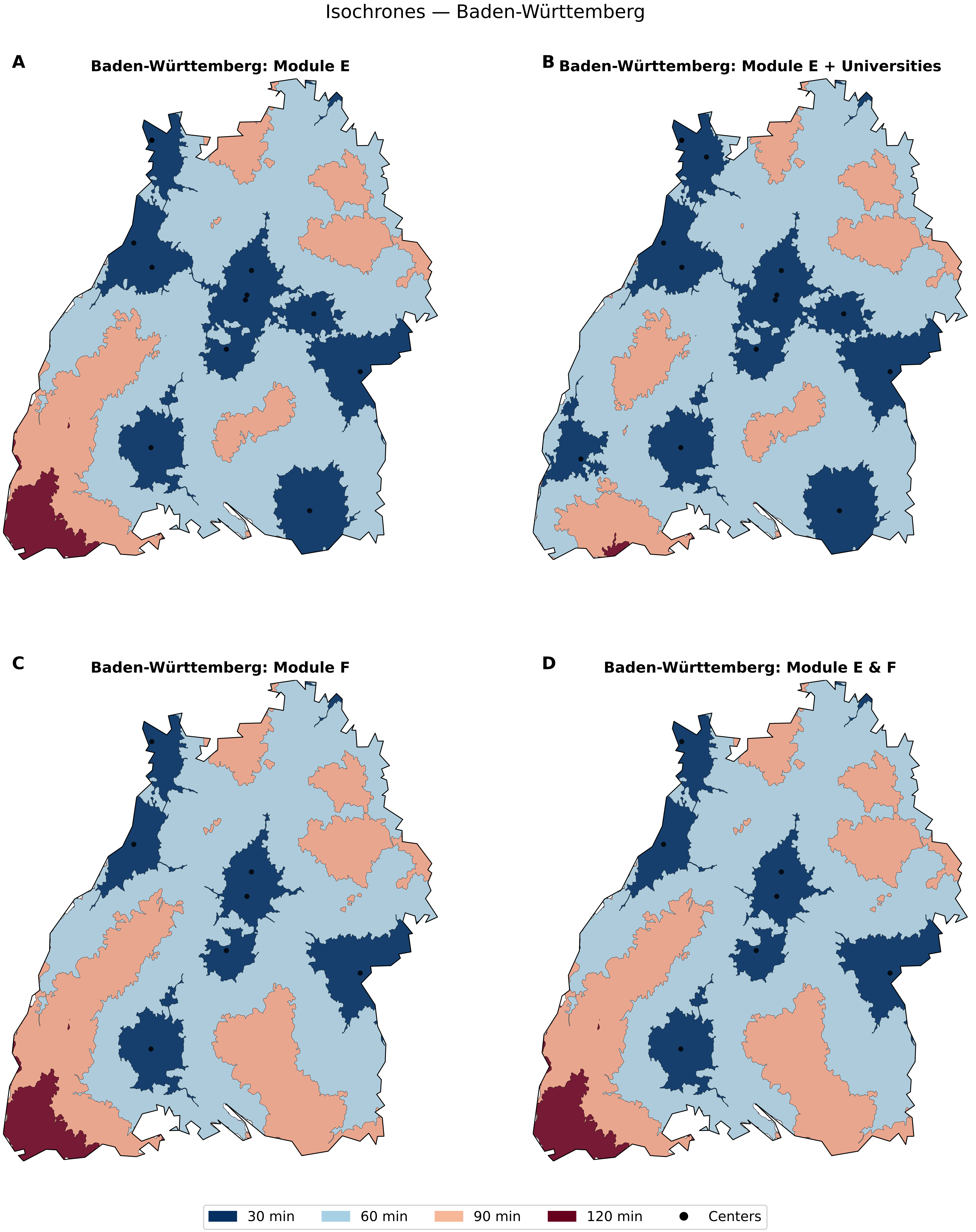
Isochrones — Baden-Württemberg
A
Baden-Württemberg: Module E
B
Baden-Württemberg: Module E + Universities
C
Baden-Württemberg: Module F
D
Baden-Württemberg: Module E & F
30 min
60 min
90 min
120 min
Centers

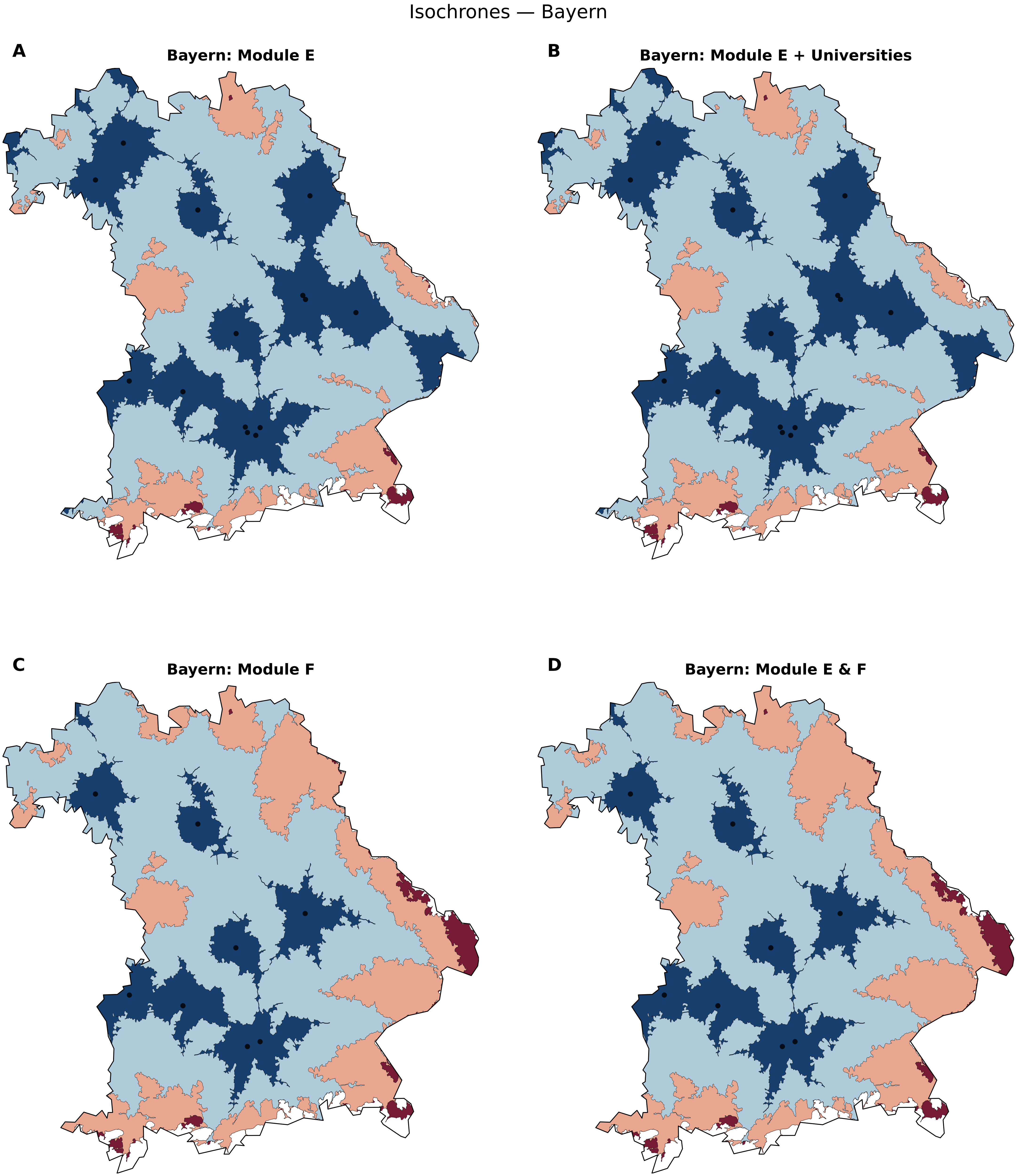
Isochrones — Bayern
A
Bayern: Module E
B
Bayern: Module E + Universities
C
Bayern: Module F
D
Bayern: Module E & F

Isochrones — Berlin

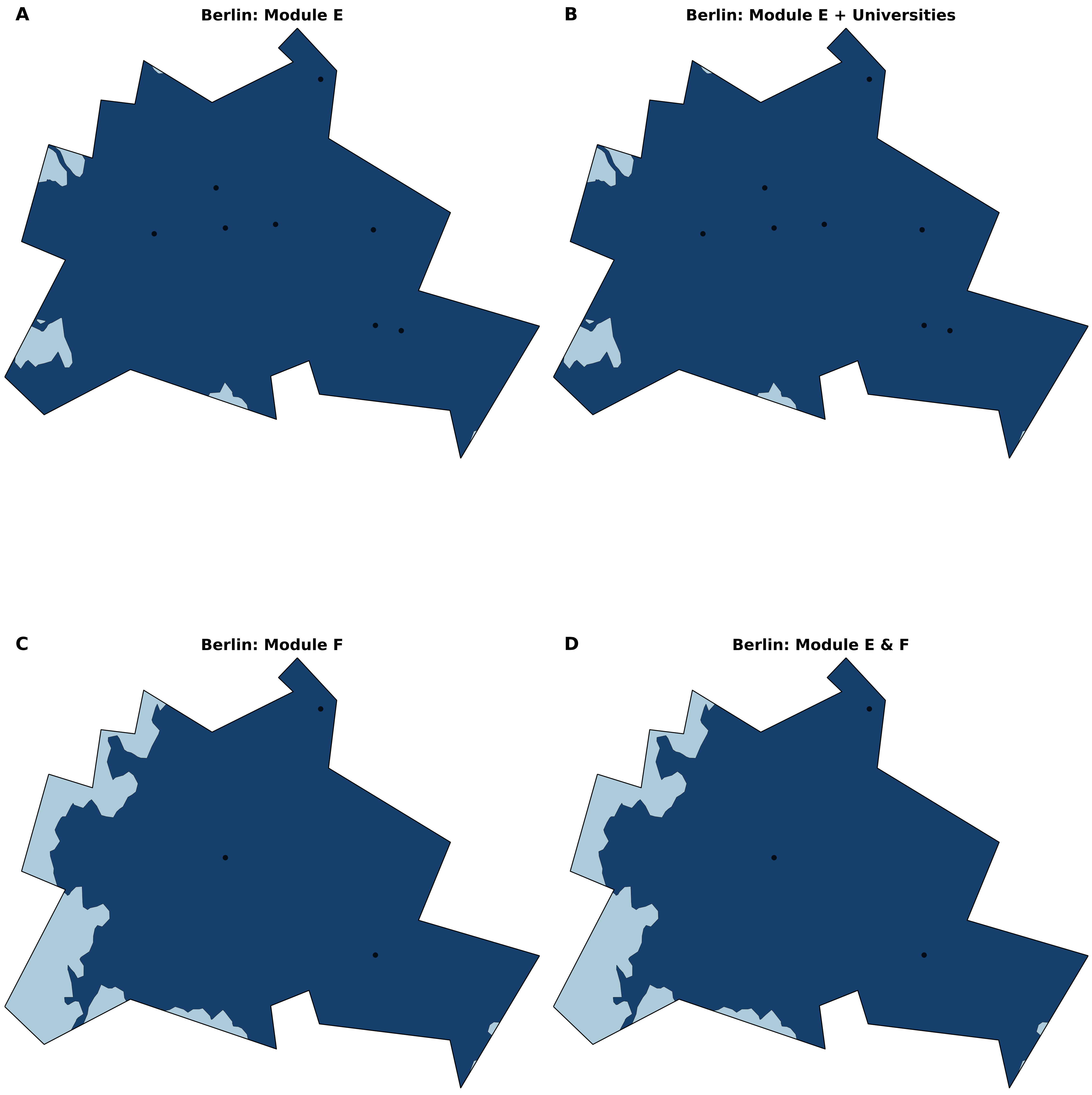

Isochrones — Brandenburg

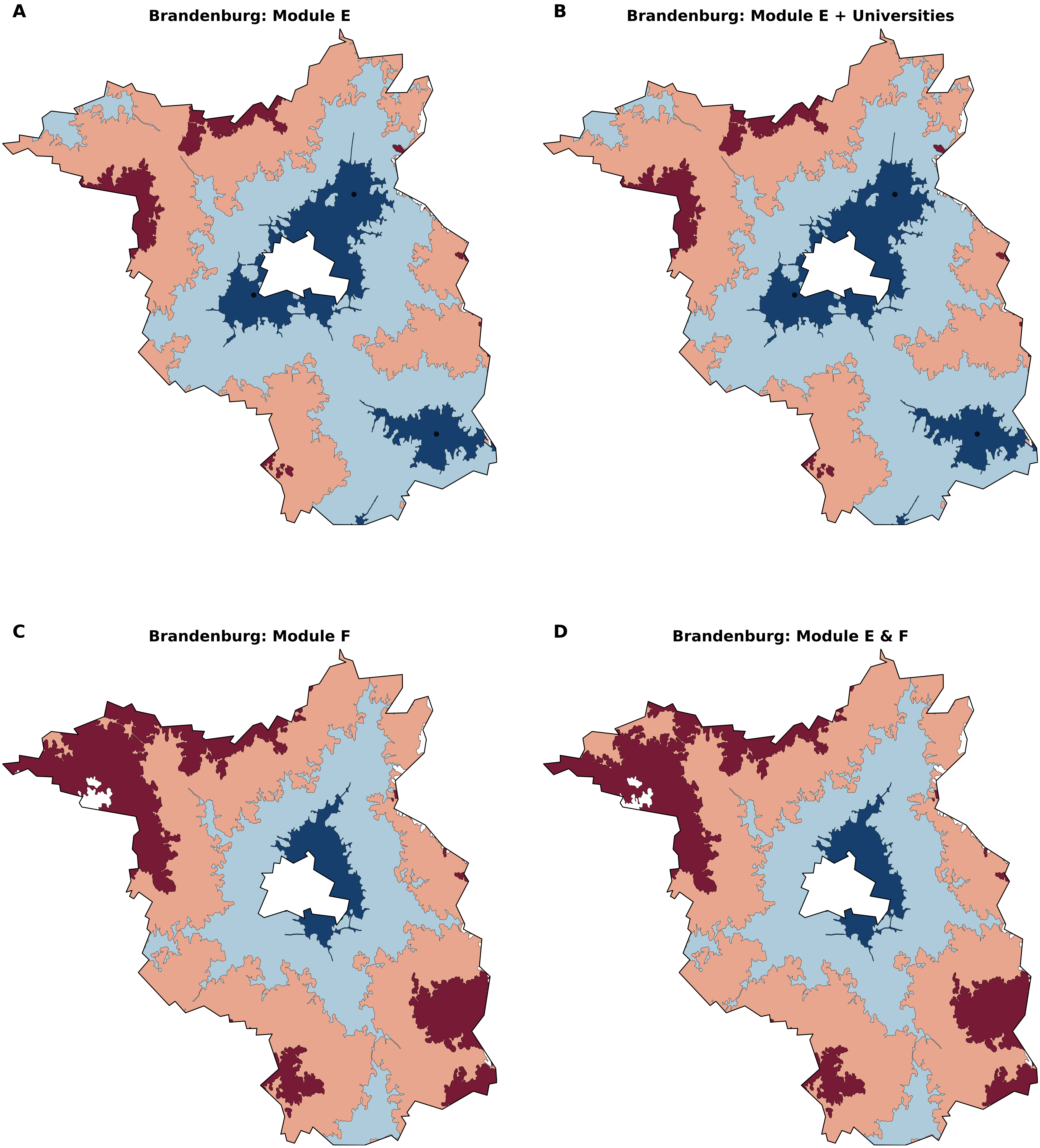

Isochrones — Bremen

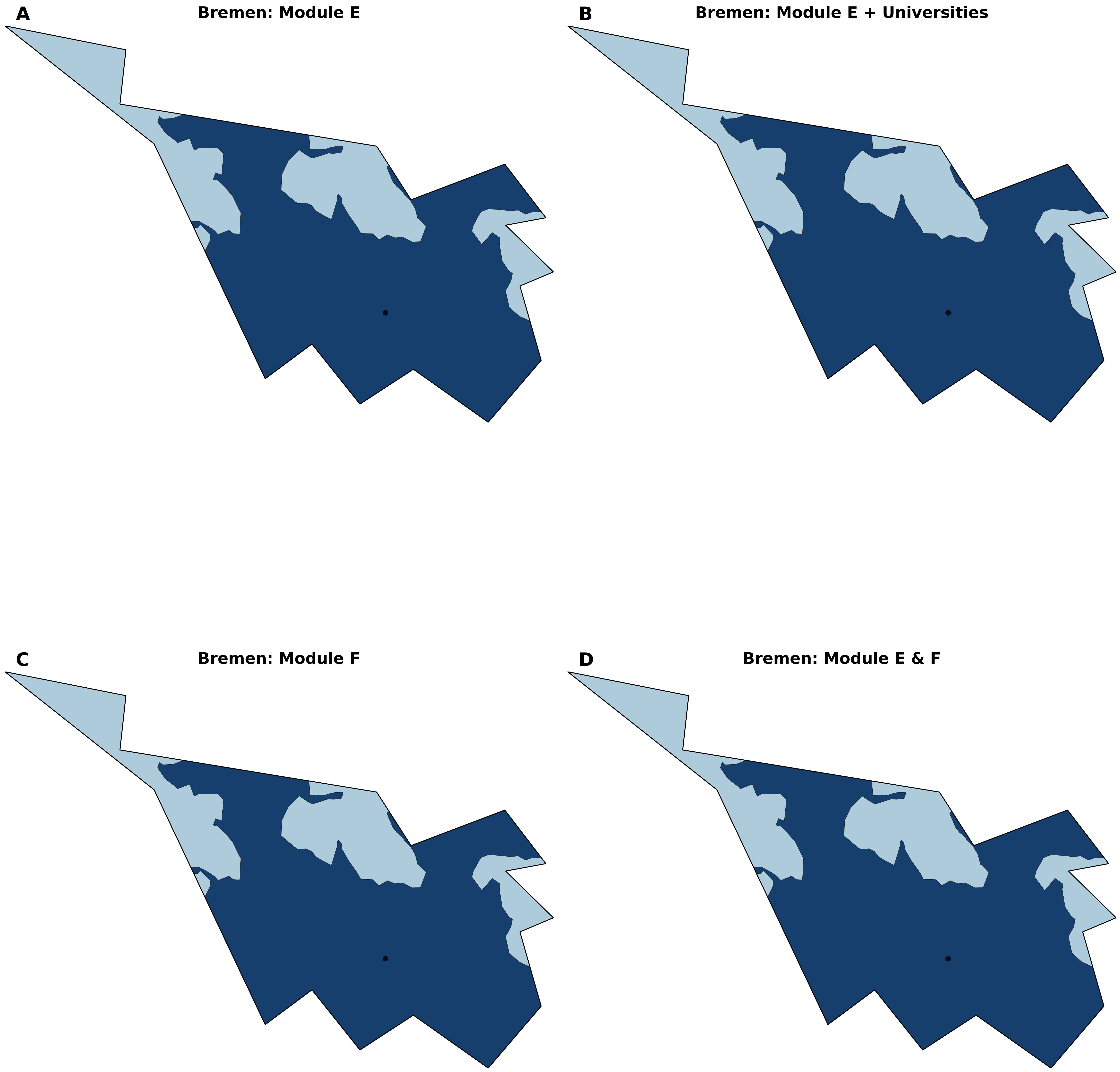

Isochrones — Hamburg

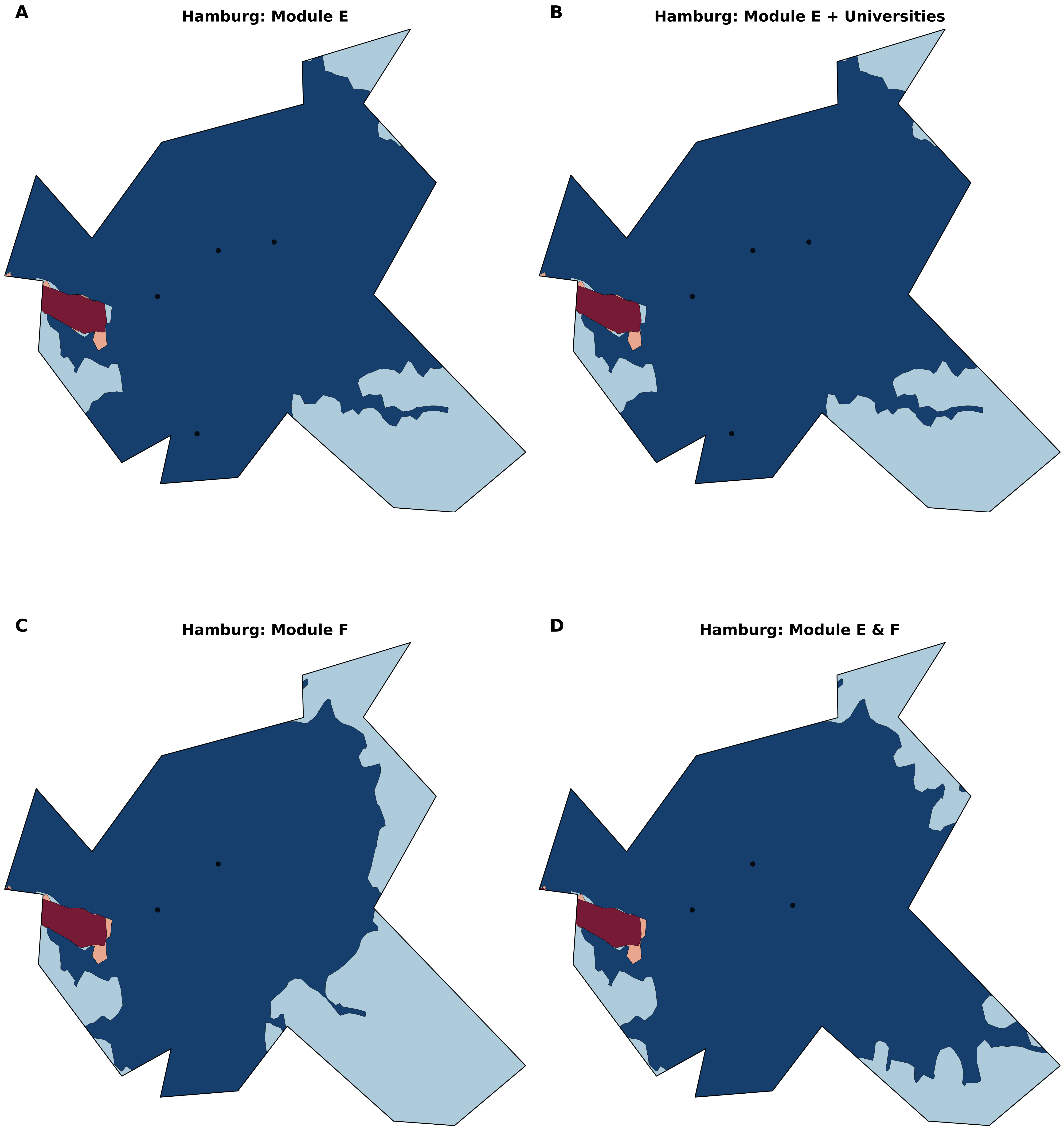

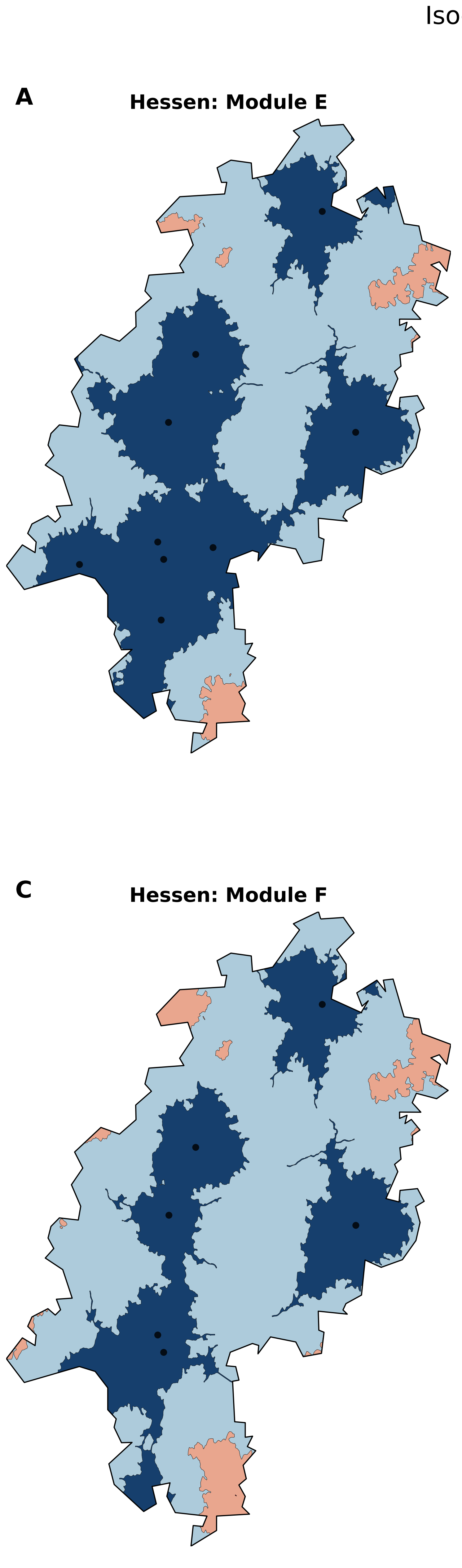
Isochrones — Hessen
A
Hessen: Module E
B
Hessen: Module E + Universities
C
Hessen: Module F
D
Hessen: Module E & F

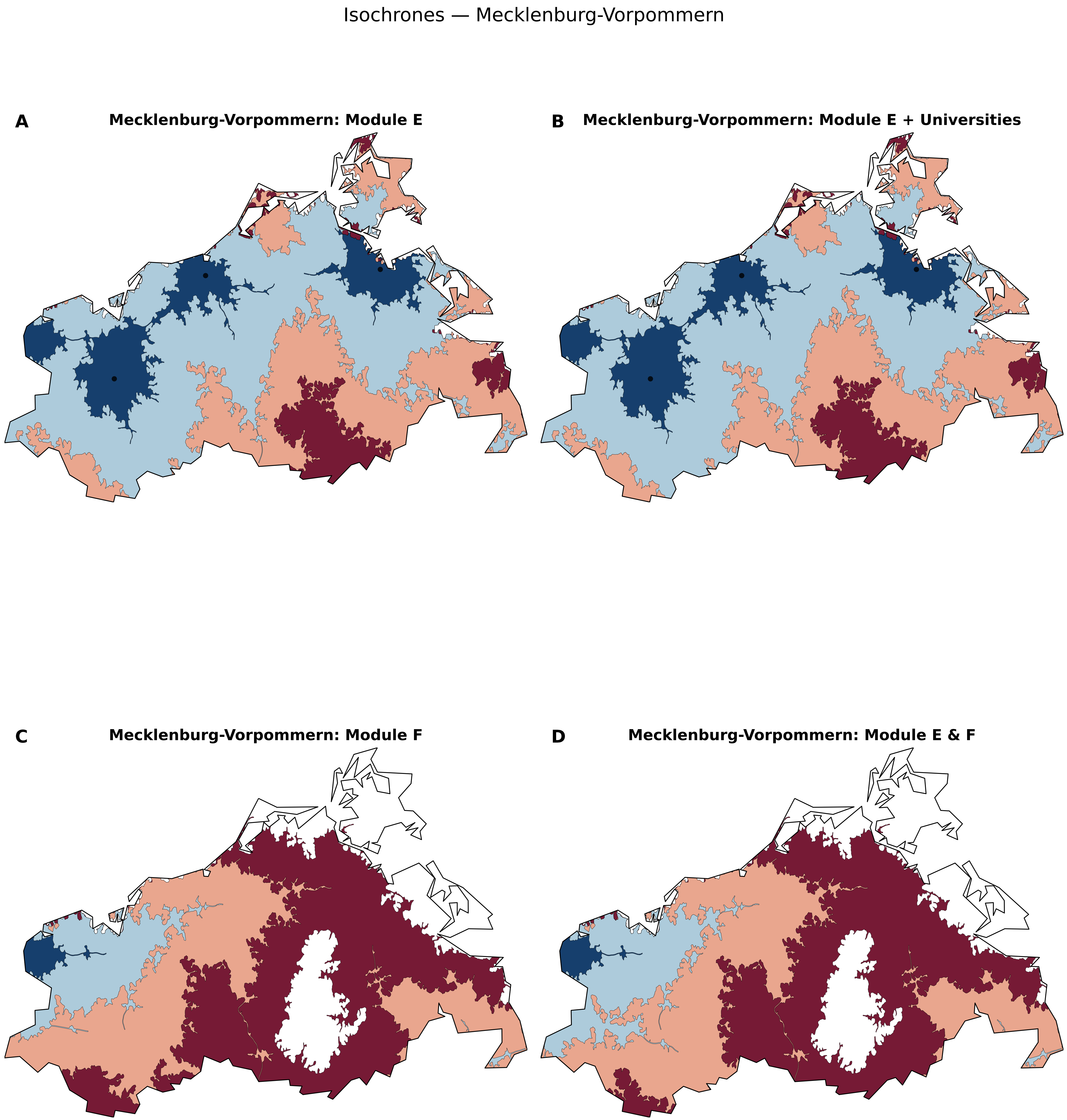
Isochrones — Mecklenburg-Vorpommern
A
Mecklenburg-Vorpommern: Module E
B
Mecklenburg-Vorpommern: Module E + Universities
C
Mecklenburg-Vorpommern: Module F
D
Mecklenburg-Vorpommern: Module E & F

Isochrones — Niedersachsen

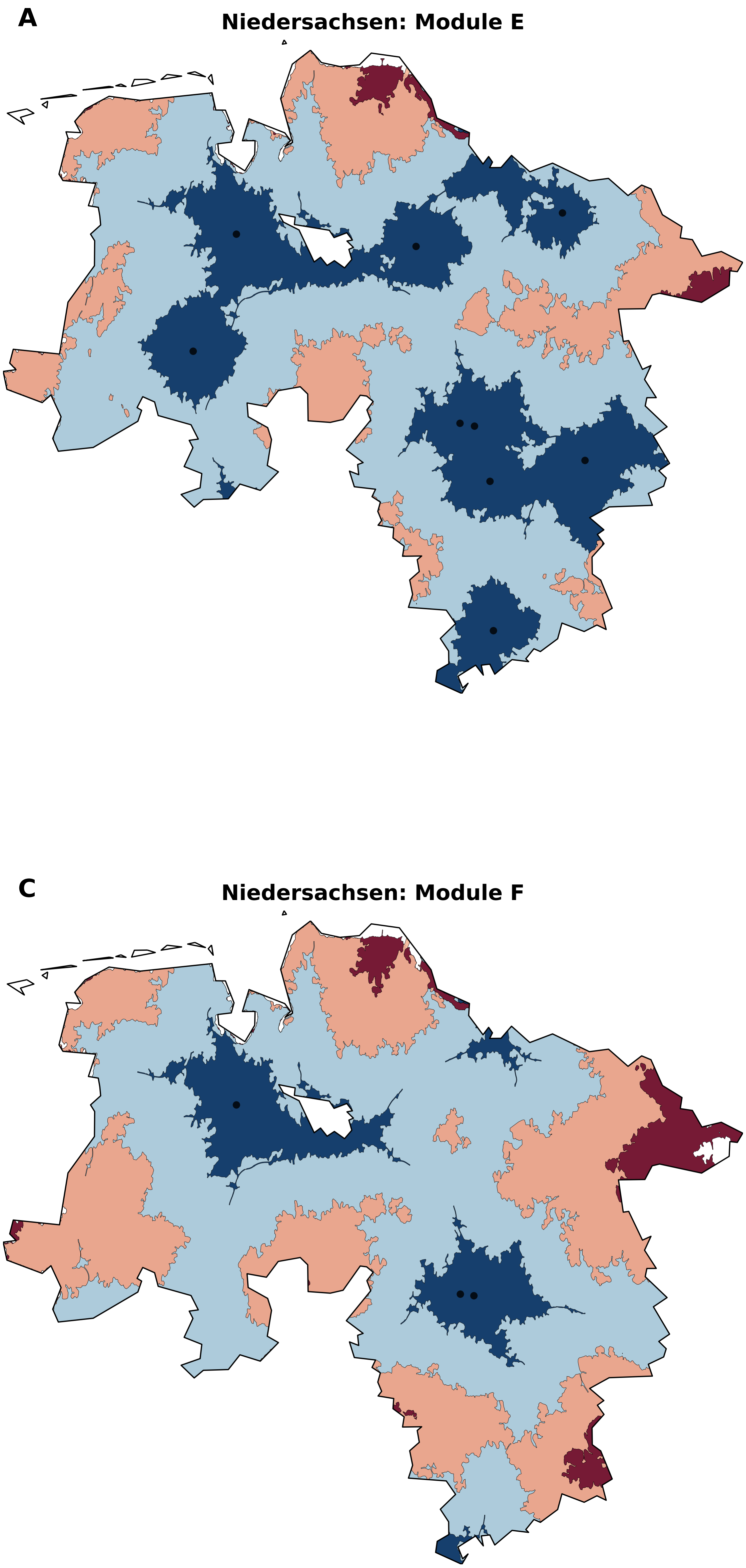

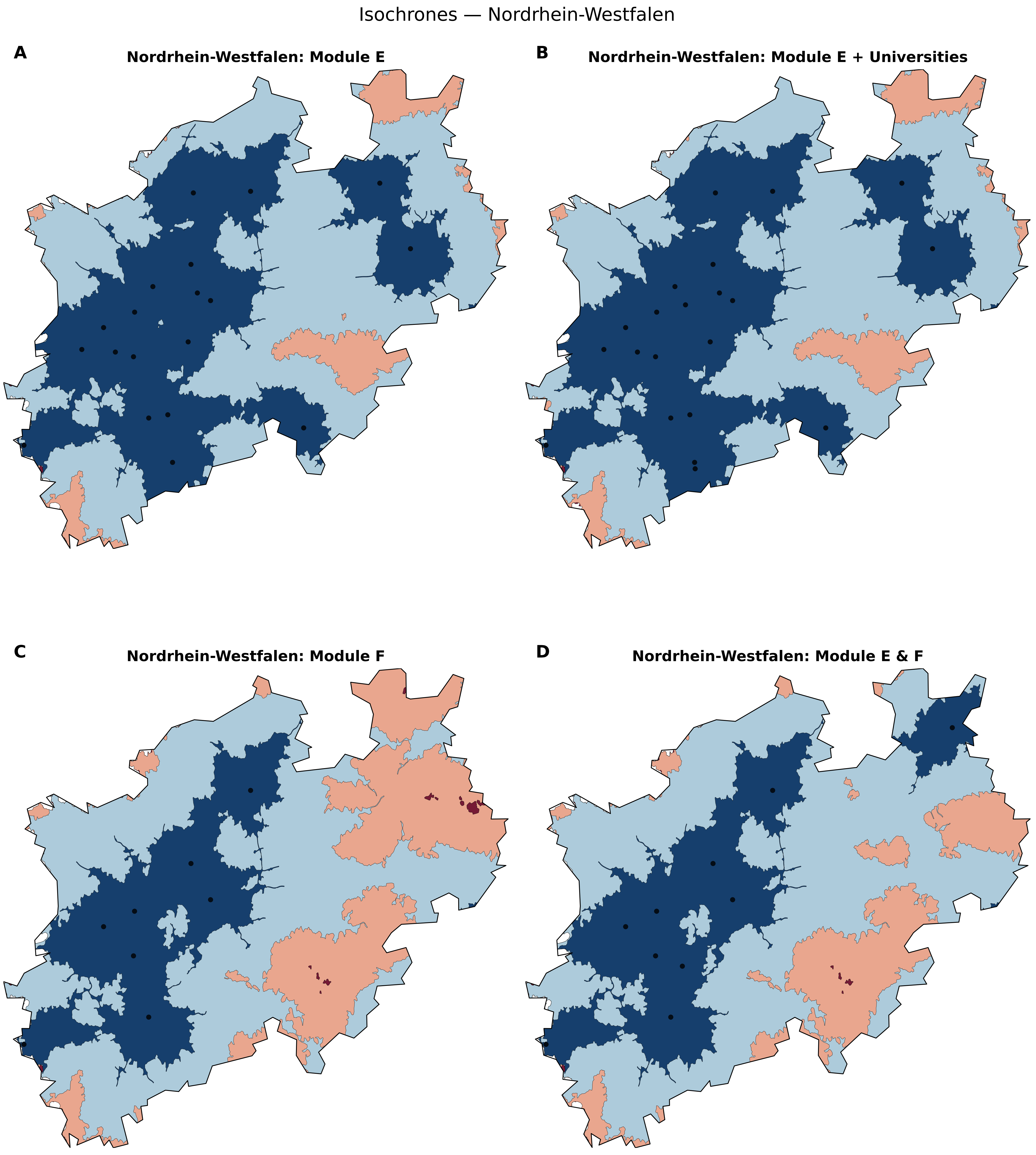

Isochrones — Nordrhein-Westfalen
A
Nordrhein-Westfalen: Module E
B
Nordrhein-Westfalen: Module E + Universities
C
Nordrhein-Westfalen: Module F
D
Nordrhein-Westfalen: Module E & F

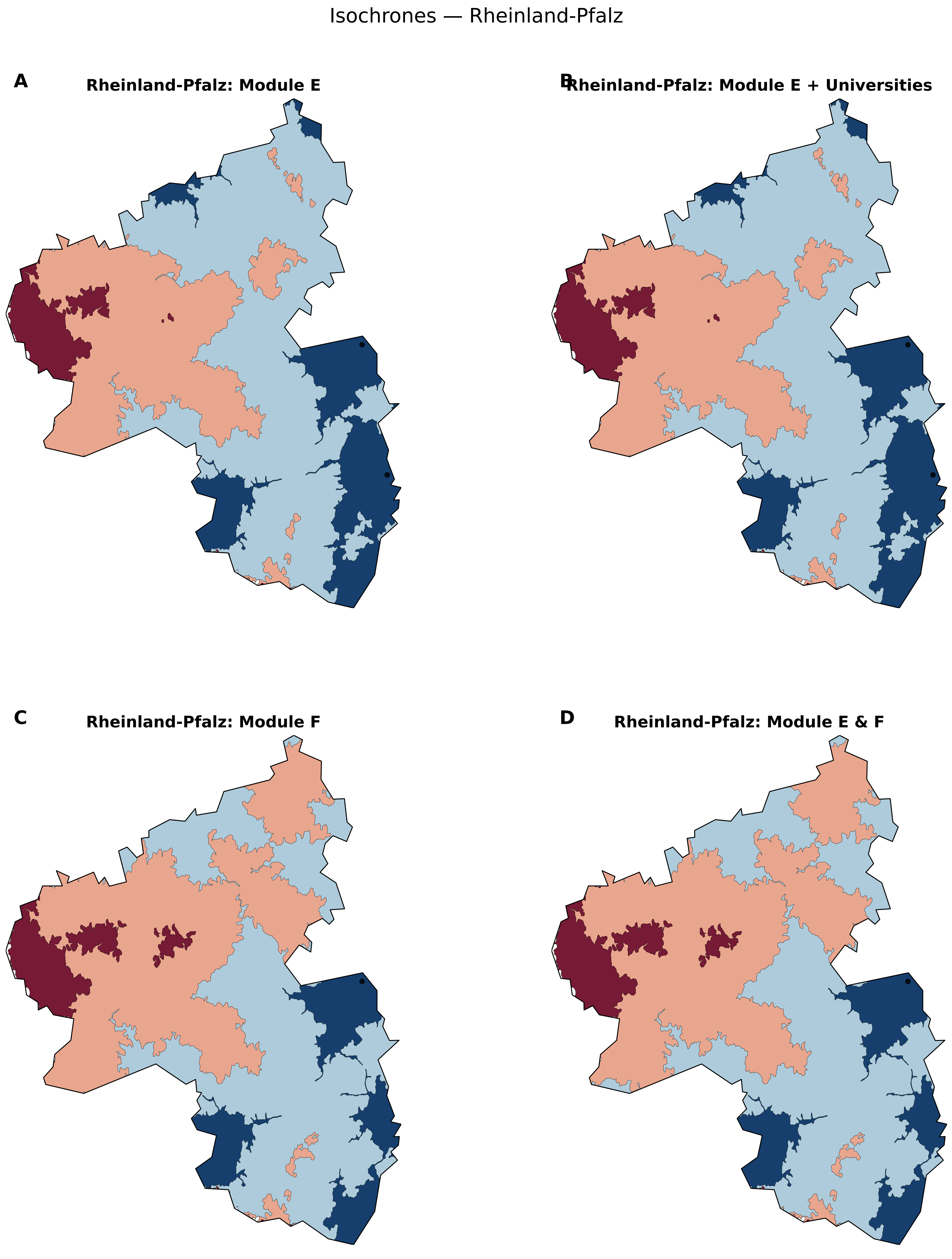
Isochrones — Rheinland-Pfalz
A
Rheinland-Pfalz: Module E
B
Rheinland-Pfalz: Module E + Universities
C
Rheinland-Pfalz: Module F
D
Rheinland-Pfalz: Module E & F

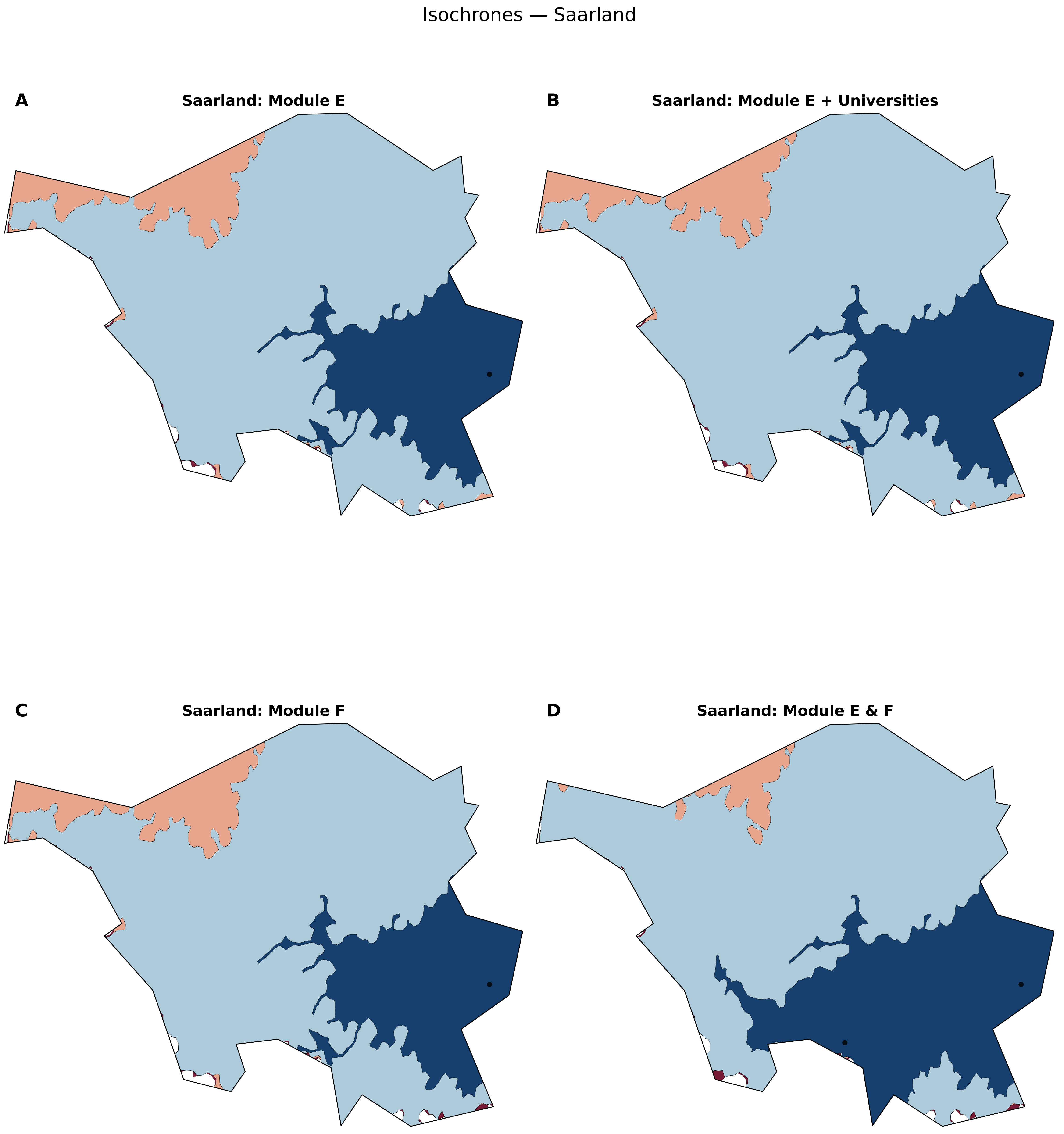
Isochrones — Saarland
A
Saarland: Module E
B
Saarland: Module E + Universities
C
Saarland: Module F
D
Saarland: Module E & F

Isochrones — Sachsen-Anhalt

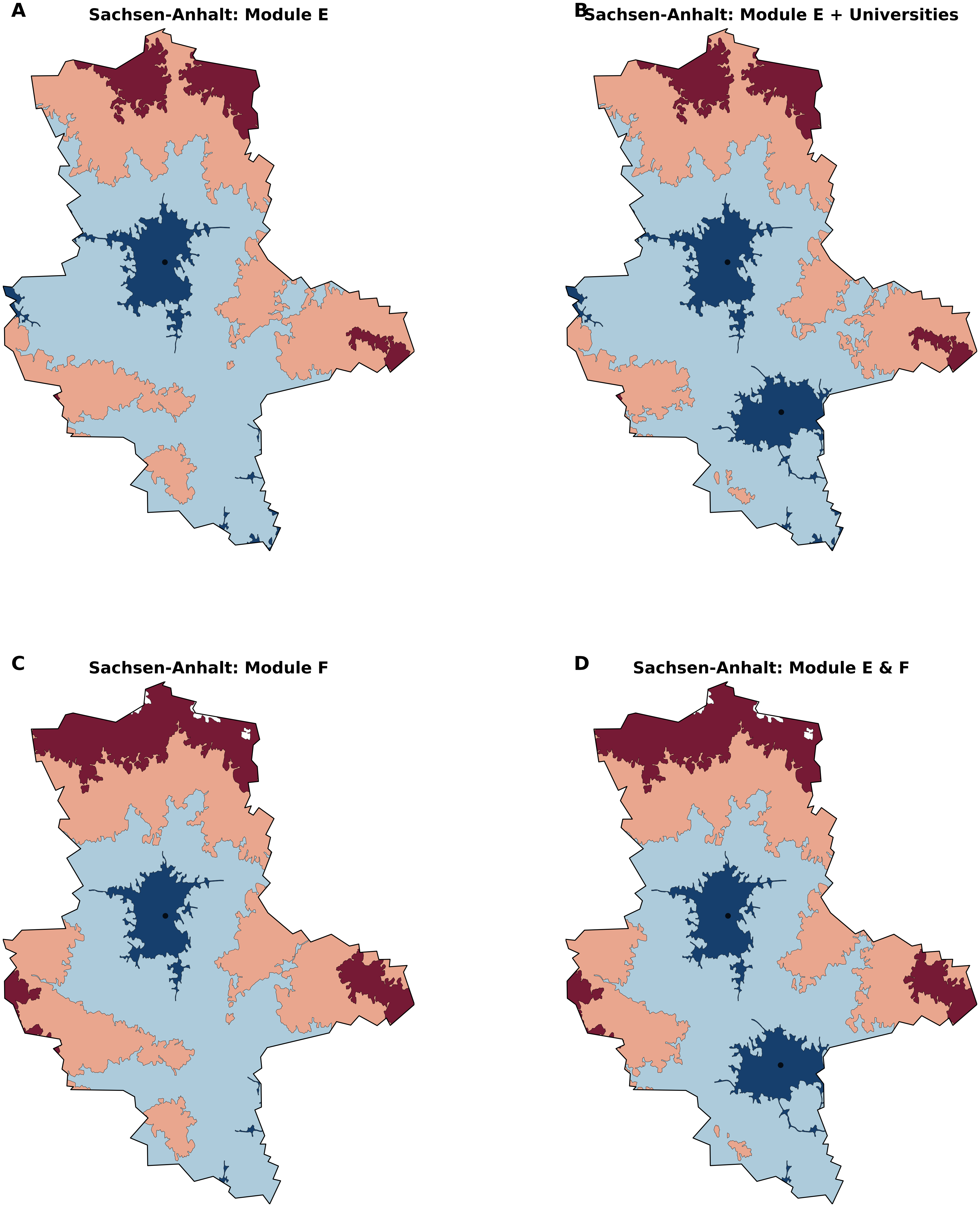

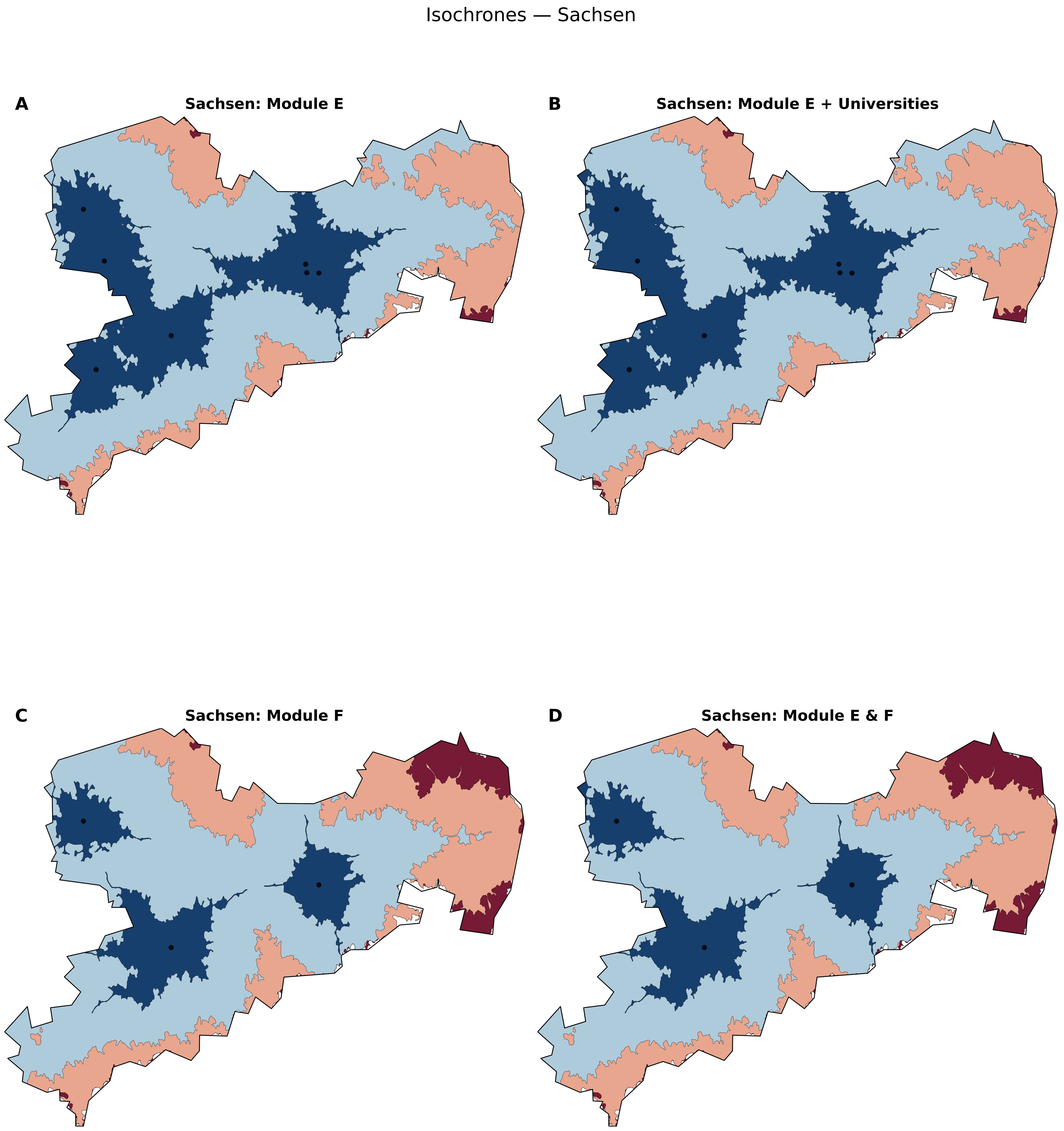
Isochrones — Sachsen
A
Sachsen: Module E
B
Sachsen: Module E + Universities
C
Sachsen: Module F
D
Sachsen: Module E & F

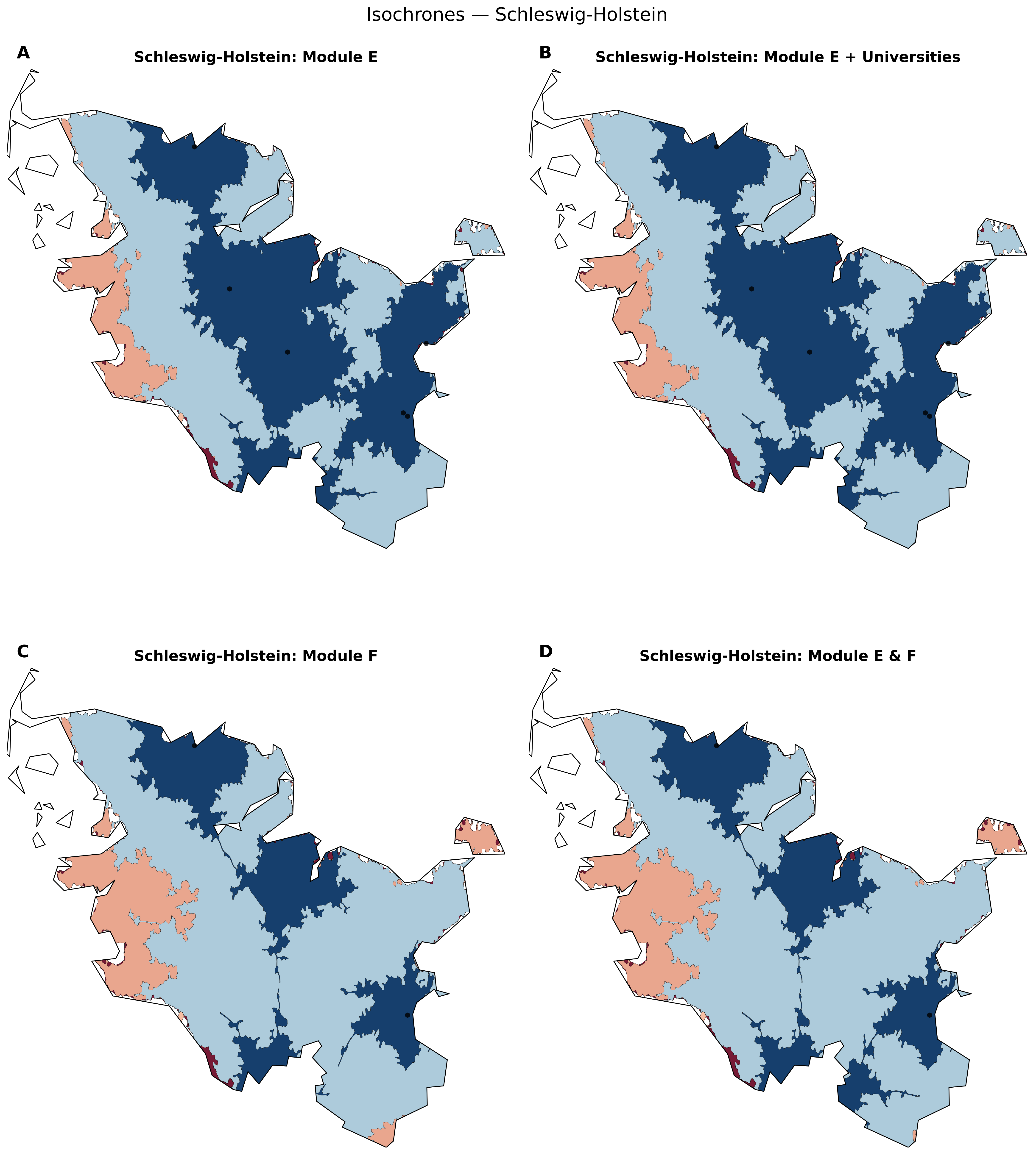
Isochrones — Schleswig-Holstein
A
Schleswig-Holstein: Module E
B
Schleswig-Holstein: Module E + Universities
C
Schleswig-Holstein: Module F
D
Schleswig-Holstein: Module E & F

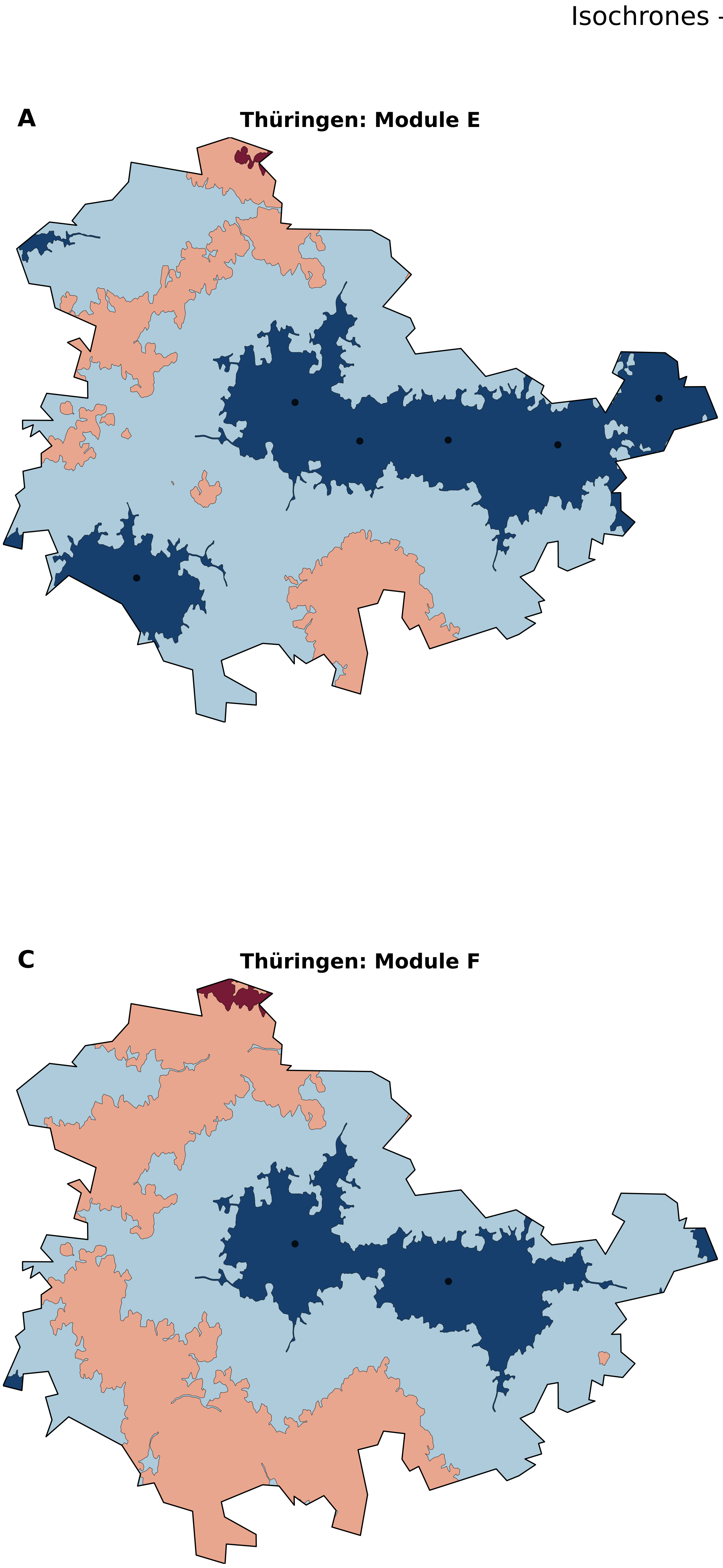
Isochrones — Thüringen
A
Thüringen: Module E
B
Thüringen: Module E + Universities
C
Thüringen: Module F
D
Thüringen: Module E & F
