# Supplementary material for: Isochrone-based Identification of Gaps in Neurovascular Care in Germany
Source: Clin Neuroradiol. 2025 Jun 26;35(4):747–53. doi: 10.1007/s00062-025-01537-0 (PMC12552351; doi:10.1007/s00062-025-01537-0)
Supplement: Supplementary file 2 — Underlying tabular representation of the data [file 62_2025_1537_MOESM2_ESM.pdf]

Population Coverage in Baden-Württemberg

Module Certification	Drive Time (min)	Covered Population	Coverage (%)
E	30	5169231	47.42
E	60	9427618	86.49
E	90	10585393	97.11
E	120	10894256	99.94
E&F	30	4095609	37.57
E&F	60	8937749	81.99
E&F	90	10584326	97.1
E&F	120	10893985	99.94
E+Universities	30	5786536	53.08
E+Universities	60	10346976	94.92
E+Universities	90	10870573	99.72
E+Universities	120	10895706	99.96
F	30	4095609	37.57
F	60	8937749	81.99
F	90	10584326	97.1
F	120	10893985	99.94

Population Coverage in Bayern

Module Certification	Drive Time (min)	Covered Population	Coverage (%)
E	30	6937931	52.13
E	60	12439770	93.47
E	90	13255971	99.6
E	120	13292562	99.88
E&F	30	5700702	42.83
E&F	60	11465555	86.15
E&F	90	13197691	99.17
E&F	120	13290966	99.87
E+Universities	30	6937931	52.13
E+Universities	60	12439770	93.47
E+Universities	90	13255971	99.6
E+Universities	120	13292562	99.88
F	30	5700702	42.83
F	60	11465555	86.15
F	90	13197691	99.17
F	120	13290966	99.87

Population Coverage in Berlin

Module Certification	Drive Time (min)	Covered Population	Coverage (%)
E	30	3600885	99.65
E	60	3613426	100.0
E	90	3613426	100.0
E	120	3613426	100.0
E&F	30	3471621	96.08
E&F	60	3613426	100.0
E&F	90	3613426	100.0
E&F	120	3613426	100.0
E+Universities	30	3600885	99.65
E+Universities	60	3613426	100.0
E+Universities	90	3613426	100.0
E+Universities	120	3613426	100.0
F	30	3471621	96.08
F	60	3613426	100.0
F	90	3613426	100.0
F	120	3613426	100.0

Population Coverage in Brandenburg

Module Certification	Drive Time (min)	Covered Population	Coverage (%)
E	30	992740	40.49
E	60	1983369	80.9
E	90	2435268	99.33
E	120	2448426	99.87
E&F	30	329806	13.45
E&F	60	1470367	59.97
E&F	90	2275430	92.81
E&F	120	2446940	99.81
E+Universities	30	992740	40.49
E+Universities	60	1983369	80.9
E+Universities	90	2435268	99.33
E+Universities	120	2448426	99.87
F	30	329806	13.45
F	60	1470049	59.96
F	90	2268211	92.52
F	120	2444960	99.73

Population Coverage in Bremen

Module Certification	Drive Time (min)	Covered Population	Coverage (%)
E	30	466925	89.12
E	60	523903	100.0
E	90	523903	100.0
E	120	523903	100.0
E&F	30	466925	89.12
E&F	60	523903	100.0
E&F	90	523903	100.0
E&F	120	523903	100.0
E+Universities	30	466925	89.12
E+Universities	60	523903	100.0
E+Universities	90	523903	100.0
E+Universities	120	523903	100.0
F	30	466925	89.12
F	60	523903	100.0
F	90	523903	100.0
F	120	523903	100.0

Population Coverage in Hamburg

Module Certification	Drive Time (min)	Covered Population	Coverage (%)
E	30	1757269	94.99
E	60	1841039	99.52
E	90	1841272	99.53
E	120	1849892	100.0
E&F	30	1795302	97.05
E&F	60	1840473	99.49
E&F	90	1841112	99.53
E&F	120	1849892	100.0
E+Universities	30	1757269	94.99
E+Universities	60	1841039	99.52
E+Universities	90	1841272	99.53
E+Universities	120	1849892	100.0
F	30	1651331	89.27
F	60	1840461	99.49
F	90	1841101	99.52
F	120	1849892	100.0

Population Coverage in Hessen

Module Certification	Drive Time (min)	Covered Population	Coverage (%)
E	30	4945596	79.07
E	60	6210124	99.29
E	90	6254410	100.0
E	120	6254410	100.0
E&F	30	3770867	60.29
E&F	60	6138602	98.15
E&F	90	6254410	100.0
E&F	120	6254410	100.0
E+Universities	30	4947857	79.11
E+Universities	60	6214369	99.36
E+Universities	90	6254410	100.0
E+Universities	120	6254410	100.0
F	30	3770867	60.29
F	60	6138602	98.15
F	90	6254410	100.0
F	120	6254410	100.0

Population Coverage in Mecklenburg-Vorpommern

Module Certification	Drive Time (min)	Covered Population	Coverage (%)
E	30	530996	36.04
E	60	1162338	78.89
E	90	1425193	96.73
E	120	1464092	99.37
E&F	30	28217	1.92
E&F	60	268711	18.24
E&F	90	834128	56.61
E&F	120	1299217	88.18
E+Universities	30	530996	36.04
E+Universities	60	1162338	78.89
E+Universities	90	1425193	96.73
E+Universities	120	1464092	99.37
F	30	28217	1.92
F	60	239733	16.27
F	90	826127	56.07
F	120	1298344	88.12

Population Coverage in Niedersachsen

Module Certification	Drive Time (min)	Covered Population	Coverage (%)
E	30	3783044	46.91
E	60	7391283	91.64
E	90	8018161	99.42
E	120	8045815	99.76
E&F	30	2153044	26.7
E&F	60	6670067	82.7
E&F	90	7968124	98.8
E&F	120	8045025	99.75
E+Universities	30	3783044	46.91
E+Universities	60	7391283	91.64
E+Universities	90	8018163	99.42
E+Universities	120	8045874	99.76
F	30	2033715	25.22
F	60	6531057	80.98
F	90	7962892	98.73
F	120	8044419	99.74

Population Coverage in Nordrhein-Westfalen

Module Certification	Drive Time (min)	Covered Population	Coverage (%)
E	30	13818877	75.18
E	60	18122721	98.59
E	90	18362761	99.9
E	120	18364260	99.91
E&F	30	11450913	62.3
E&F	60	17602616	95.76
E&F	90	18361213	99.89
E&F	120	18361883	99.89
E+Universities	30	13819939	75.19
E+Universities	60	18122872	98.59
E+Universities	90	18364459	99.91
E+Universities	120	18365874	99.92
F	30	10844905	59.0
F	60	16256062	88.44
F	90	18343971	99.8
F	120	18361840	99.89

Population Coverage in Rheinland-Pfalz

Module Certification	Drive Time (min)	Covered Population	Coverage (%)
E	30	1559406	37.44
E	60	3392773	81.45
E	90	4112355	98.73
E	120	4164773	99.99
E&F	30	1109221	26.63
E&F	60	2782706	66.81
E&F	90	4102568	98.49
E&F	120	4164825	99.99
E+Universities	30	1559406	37.44
E+Universities	60	3392773	81.45
E+Universities	90	4112355	98.73
E+Universities	120	4164795	99.99
F	30	1109221	26.63
F	60	2777873	66.69
F	90	4102064	98.48
F	120	4164769	99.99

Population Coverage in Saarland

Module Certification	Drive Time (min)	Covered Population	Coverage (%)
E	30	292982	30.06
E	60	952137	97.69
E	90	973120	99.84
E	120	973311	99.86
E&F	30	534439	54.83
E&F	60	970075	99.53
E&F	90	973125	99.84
E&F	120	973331	99.86
E+Universities	30	292982	30.06
E+Universities	60	952137	97.69
E+Universities	90	973120	99.84
E+Universities	120	973381	99.87
F	30	292982	30.06
F	60	952137	97.69
F	90	973023	99.83
F	120	973175	99.85

Population Coverage in Sachsen-Anhalt

Module Certification	Drive Time (min)	Covered Population	Coverage (%)
E	30	390678	18.58
E	60	1729516	82.23
E	90	2076805	98.74
E	120	2103214	100.0
E&F	30	713278	33.91
E&F	60	1703328	80.99
E&F	90	2029978	96.52
E&F	120	2103054	99.99
E+Universities	30	739703	35.17
E+Universities	60	1819682	86.52
E+Universities	90	2076805	98.74
E+Universities	120	2103214	100.0
F	30	368409	17.52
F	60	1611218	76.61
F	90	2028212	96.43
F	120	2103044	99.99

Population Coverage in Sachsen

Module Certification	Drive Time (min)	Covered Population	Coverage (%)
E	30	2499323	60.69
E	60	3803157	92.34
E	90	4109012	99.77
E	120	4116876	99.96
E&F	30	2077276	50.44
E&F	60	3572383	86.74
E&F	90	4034461	97.96
E&F	120	4116437	99.95
E+Universities	30	2504137	60.8
E+Universities	60	3803157	92.34
E+Universities	90	4109012	99.77
E+Universities	120	4116905	99.96
F	30	2075496	50.39
F	60	3572383	86.74
F	90	4034461	97.96
F	120	4116437	99.95

Population Coverage in Schleswig-Holstein

Module Certification	Drive Time (min)	Covered Population	Coverage (%)
E	30	1757735	63.43
E	60	2659863	95.99
E	90	2733794	98.65
E	120	2737714	98.79
E&F	30	1354979	48.9
E&F	60	2582725	93.2
E&F	90	2729585	98.5
E&F	120	2736655	98.76
E+Universities	30	1757735	63.43
E+Universities	60	2659863	95.99
E+Universities	90	2733794	98.65
E+Universities	120	2737714	98.79
F	30	1246089	44.97
F	60	2574573	92.91
F	90	2729264	98.49
F	120	2736444	98.75

Population Coverage in Thüringen

Module Certification	Drive Time (min)	Covered Population	Coverage (%)
E	30	920202	45.33
E	60	1870098	92.12
E	90	2030143	100.0
E	120	2030174	100.0
E&F	30	649057	31.97
E&F	60	1592914	78.46
E&F	90	2029938	99.99
E&F	120	2030174	100.0
E+Universities	30	920202	45.33
E+Universities	60	1873036	92.26
E+Universities	90	2030143	100.0
E+Universities	120	2030174	100.0
F	30	649057	31.97
F	60	1589056	78.27
F	90	2029861	99.98
F	120	2030174	100.0
